# Recommended Quantities of Emollients and Topical Corticosteroids for Eczema Management: A Comprehensive UK Survey of Hospital Guidance

**DOI:** 10.1111/cea.70393

**Published:** 2026-07-14

**Authors:** Lucy Mackie, Sorcha O'Sullivan, Marcus Tan, Shahara Ali, Alya Abdul Wahab, Leila Ferguson, Michael R. Perkin

**Affiliations:** ^1^ Department of Dermatology St George's University Hospitals NHS Foundation Trust London UK; ^2^ Department of Pharmacology City St. George's University of London London UK; ^3^ Department of Population Health & Policy City St. George's, University of London London UK

## Abstract

- Advice regarding the quantity of emollient and steroid creams to be applied varies substantially between hospitals.
- Greater standardisation of quantitative advice may improve confidence, adherence, and treatment outcomes.

To the Editor,

Clear guidance on the quantity and regimen of topical treatments is essential for patients with eczema, yet inconsistency persists in clinical practice. We report findings from a UK‐wide Freedom of Information request sent to 156 National Health Service (NHS) Trusts on the 17/04/2024 exploring how dermatology services quantify advice on topical corticosteroid and emollient use. An NHS Trust consists of one or more hospitals providing services to a geographical area. The questions were: ‘What advice does your hospital's dermatology department issue to patients with eczema regarding: (1) the amount of moisturiser, and (2) the amount of steroid cream/ointment, that should be applied to patients?’

We received replies from 133 Trusts, within 20 working days. Three refused the FOI request, 14 did not have a dermatology service, and 4 provided no data, leaving 112 responses for analysis (Table 1). Many respondents emphasised that prescribing is individualised.

**TABLE 1:** Summary responses from 112 trusts for quantification of topical emollients and topical corticosteroids.

	Topical corticosteroids *n* (%)	Topical emollients *n* (%)
Quantities given	22 (19.6%)	55 (49.1)
Qualitative terms used	15 (13.3%)	19 (17.0)
Frequency mentioned	32 (28.6)	40 (35.7)
Leaflet	79 (70.5)	76 (67.9)
Finger tip units—Direct^a^	50 (44.6)	NA
Finger tip units—Indirect^b^	43 (39.4)	NA

*Note:* Trusts could give positive replies for more than one option.

^a^ Direct means FTU were explicitly mentioned in the Trust response.

^b^ Indirect means FTU were mentioned in a leaflet or link (to BNF or NICE guidance) provided by the Trust.

Advice on quantities of topical corticosteroids most commonly referred to finger‐tip units (FTUs) [1, 2]. Fifty services explicitly stated that they use FTUs in prescribing, while 19 referenced FTUs within departmental patient information leaflets. Eleven services stated that they follow National Institute for Health and Care Excellence (NICE) or British National Formulary (BNF) guidance, and 12 provided links to external leaflets, most commonly the British Association of Dermatologists (BAD) topical corticosteroid leaflet [3], all of which reference FTUs. Eleven services correctly stated that one FTU equates to approximately 0.5 g [1, 2]. This covers the size of two hand areas (adult palms plus fingers) [4]. Two services explicitly referred to BNF/NICE quantity tables outlining amounts required per body site daily over a two‐week period (about half for a child): face and neck 15–30 g, hands 15–30 g, scalp 15–30 g, arms 30–60 g, legs 100 g, trunk 100 g and groin and genitalia 15–30 g [5]. Eleven services provided other specific prescription quantities, which varied considerably. Qualitative descriptors were also used, including to apply treatment until the skin ‘glistens’ (7), ‘shines’ (2), in a ‘thin layer’ (4), or ‘sparingly’ (2). The word ‘sparingly’ is also mentioned in NICE guidance [5].

Considerable variation was observed in advice regarding frequency and duration of use. NICE guidance supports initial once‐daily use on the body for 7–14 days matching potency to severity, application for 48 h after flare resolution, and intermittent or step‐down maintenance strategies [5]. Fifteen services recommended once‐daily application, 8 advised once or twice daily, and 5 advised twice daily. Eleven described tapering by reducing frequency, 3 by reducing potency, 2 suggested either approach, and 2 suggested both. These regimens were rarely consistent between services. Advice on when to stop treatment also differed, with some recommending cessation once the eczema cleared, others advising tailing off, stopping 1 week after clearance, or continuing for 2 days after clearance. A small number of respondents described twice‐weekly or ‘weekend’ maintenance therapy for patients with frequent flares. NICE defines frequent flares as 2–3 per month [5]. Overall, 79 services provided patients with at least one leaflet about topical steroid use: 45 from the BAD [3], 29 unique to a service, and others from the National Eczema Society, Eczema Outreach Support and Eczema Care Online.

Advice on topical emollient use was similarly variable. Fifty‐five services provided specific quantities in grams, most commonly recommending 250–500 g of emollient per week for children, often citing NICE guidance [5]. Five provided site‐specific quantities, such as 15–30 g for the face and neck, 30–60 g for arms, or 100 g for legs or trunk. Qualitative descriptive terms were widely used, such as apply ‘liberally’ (9), ‘generously’ (3), ‘copiously’ (2), until ‘glistening’ (1), ‘adequately hydrated’ (1) or ‘slightly greasy’ (1), in a ‘thick layer’ (1), or in ‘plenty’ (1). Twenty different frequencies of application were suggested, with the most common being four times a day (6). Eighteen services provided no quantitative or descriptive guidance. Seventy‐six services provided patient information leaflets regarding emollients, most commonly from the BAD [6], which do not include specific quantities.

Atopic eczema affects around one in five children and one in 10 adults in the UK. While biologic therapies have transformed the management of severe disease, topical treatments remain the cornerstone of care for mild to moderate eczema and an essential adjunct in more severe cases. Underuse of topical treatments is common [7], conflated by growing patient and family concerns about side effects from overuse [8].

Our findings demonstrate widespread reliance on FTUs for topical corticosteroids, supported by national guidance [5], but also reveal substantial variation in regimens and frequent use of qualitative descriptors for both steroids and emollients. Such descriptors are open to interpretation and may contribute to inconsistent use and reduced patient confidence. The wide variability in advice between healthcare providers also likely further reduces confidence and trust. Such descriptors also make it hard to quantify when under or overuse may have occurred.

These findings align with a 2022 Cochrane review [9], which highlighted patient confusion regarding how best to use topical corticosteroids and identified limited high‐quality evidence guiding duration and no evidence guiding tapering strategies. NICE guidance makes suggestions on these strategies [5], yet our data suggest that translation of this into consistent clinical advice remains variable; this may be in part due to the limited evidence base.

Our results represent the responses submitted. In our clinical experience, many patients deny receiving any quantification advice, whether because leaflets haven't been read or recommended websites visited. However, clearly greater standardisation of quantitative advice, alongside clear patient‐facing information, is a first step towards helping improve understanding, adherence, and outcomes for people living with eczema.

## Author Contributions

**Lucy Mackie:** data curation, formal analysis, validation, visualisation, writing – original draft, writing – review and editing. **Sorcha O'Sullivan:** data curation, formal analysis, validation, visualisation, writing – review and editing. **Marcus Tan:** data curation, formal analysis, investigation, methodology, writing – review and editing. **Shahara Ali:** data curation, formal analysis, investigation, methodology, writing – review and editing. **Alya Abdul Wahab:** supervision, writing – review and editing. **Leila Ferguson:** supervision, writing – review and editing. **Michael R. Perkin:** conceptualisation, data curation, formal analysis, investigation, methodology, project administration, resources, supervision, validation, visualisation, writing – original draft, writing – review and editing.

## Funding

The authors have nothing to report.

## Ethics Statement

The authors have nothing to report.

## Conflicts of Interest

The authors declare no conflicts of interest.

## Data Availability

The data that supported the findings of this survey are openly available at City St George's FigShare, data repository (https://city.figshare.com/).

## References

[1] C. C. Long and A. Y. Finlay , “The Finger‐Tip Unit—A New Practical Measure,” Clinical and Experimental Dermatology 16, no. 6 (1991): 444–447. 10.1111/j.1365-2230.1991.tb01232.x 1806320

[2] C. C. Long , C. M. Mills , and A. Y. Finlay , “A Practical Guide to Topical Therapy in Children,” British Journal of Dermatology 138, no. 2 (1998): 293–296. 10.1046/j.1365-2133.1998.02077.x 9602877

[3] British Association of Dermatologists , “Topical Corticosteroids,” (2012), https://www.bad.org.uk/pils/topical‐corticosteroids.

[4] C. C. Long , A. Y. Finlay , and R. W. Averill , “The Rule of Hand: 4 Hand Areas = 2 FTU = 1 g,” Archives of Dermatology 128, no. 8 (1992): 1129–1130. 1497374

[5] National Institute for Clinical Excellence (NICE) , “Atopic Eczema in Under 12s: Diagnosis and Management,” (2007). 34101396

[6] British Association of Dermatologists , “Emollient Use in Skin Conditions,” (2023), https://www.bad.org.uk/pils/emollient‐use‐in‐skin‐condition.

[7] H. Kv , A. Bohnen , S. Macneill , et al., “Topical Treatment Use and Under‐Use in Childhood Eczema: Secondary Analysis of RCTs and Cohort Data in the UK and The Netherlands,” BJGP Open (2026): BJGPO.2026.0005. 10.3399/BJGPO.2026.0005 41760286

[8] Medicines & Healthcare products Regulatory Agency , “Topical Steroid Withdrawal Reactions: A Review of the Evidence,” (2021), https://www.gov.uk/government/publications/topical‐steroid‐withdrawal‐reactions‐a‐review‐of‐the‐evidence/topical‐steroid‐withdrawal‐reactions‐a‐review‐of‐the‐evidence.

[9] S. J. Lax , J. Harvey , E. Axon , et al., “Strategies for Using Topical Corticosteroids in Children and Adults With Eczema,” Cochrane Database of Systematic Reviews 3 (2022). 10.1002/14651858.CD013356.pub2 PMC8916090 35275399

